# Filter Masks during the Second Phase of SARS-CoV-2: Study on Population

**DOI:** 10.3390/ijerph20032360

**Published:** 2023-01-29

**Authors:** Enzo Cumbo, Giuseppe Gallina, Pietro Messina, Giuseppe Alessandro Scardina

**Affiliations:** Department of Surgical Oncological and Stomatological Disciplines, University of Palermo, Via Del Vespro 129, 90127 Palermo, Italy

**Keywords:** COVID-19, masks, information, incorrect behavior

## Abstract

During the SARS-CoV-2 pandemic, the most common countermeasure are the use of masks, which are supposed to filter inhaled and exhaled air to reduce the spread of the virus. The masks, which are medical devices, must be used by providing appropriate instructions for correct use. This study, which examined the population during the advanced stage of the pandemic, highlighted a substantial improvement in the management and choice of masks, even though the information disseminated to the population probably should be even more detailed and precise in order to avoid incorrect behavior that could compromise the effectiveness of these devices; in fact a high percentage of subjects had behaviors that can facilitate the spread of the virus, such as the continuous attempts to correct the incorrect positioning of the mask on the face or the need to move it because it is annoying.

## 1. Introduction

The COVID-19 pandemic is nowadays a fairly well-known and studied disease for which it is understood that it is necessary to limit the airborne spread of this virus in every way.

One of the solutions most adopted by governments was the lockdown, through which the citizens of most nations were forced to stay inside their homes for months, completely canceling all social relations. Another countermeasure was that of “social distancing”, which literally prevented non-cohabiting subjects, when they were outside their homes, from staying close to each other, thus avoiding the gatherings that would facilitate the infections. Mass vaccination further helped the fight against COVID-19 and strengthened our immune defenses against this microorganism to which we had no immunity whatsoever. Other concepts, of which attempts were made in all countries to disseminate information, are concepts relating to personal hygiene, and above all, hygiene of the hands, by numerous advising commercials and demonstration videos; to this was also added the ubiquitous availability of disinfectant gels present in most places accessible to the public, such as stores. Finally, the imposition of the use of filter masks by governments of most of the entire planet represented another fundamental decision with the intention of limiting the circulation of the virus as much as possible, especially when citizens were confined in spaces that were poorly ventilated [1,2,3,4].

The massive use of filter masks, in normal daily activities, helped to contain the contagion among the population because the transmission of this virus occurs mainly by air [5,6,7,8,9].

However, we must consider the way in which the mask is managed by people who, when the pandemic began to spread, were not adequately informed on how to behave [10,11].

That lack of precise information certainly led the population to make serious mistakes using those masks; they were often worn of not covering their nose and mouth correctly. All this led to a reduction in the filtering efficiency of this device, reducing its effectiveness in a historical moment when the lack of mass vaccination prompted doctors to try to reduce the airborne spread of the virus by any means [12,13]. 

Most likely, the breathing difficulties caused by this filter led the population not to wear it correctly; in fact, the FFP2, and above all, the FFP3 masks, were little used at the beginning of the pandemic precisely because they made breathing difficult, and also due to their shape, which is made to make the edges adhere well to the face. These difficulties, together with the fact that the population was not at all used to circulating wearing such medical devices, often led citizens to leave the mask itself non-adherent to the skin, letting unfiltered air pass both during inspiration and expiration, invalidating its function. 

In addition, people who are particularly intolerant of the breathing difficulties caused by filtering masks chose to wear those with valves that filter only the inhaled air, leaving the exhaled air, which could be potentially infected, to escape unfiltered. 

To this is added an incorrect management, such as touching the mask repeatedly because it is a source of discomfort or trying to position it better on the face. These incorrect behaviors can make the masks even more ineffective or make them a dangerous vehicle for the spread of the disease itself.

Nowadays, in order to drastically reduce the problems mentioned, a massive information campaign was organized in all countries, which tried to spread the correct use of this important medical device.

However, it is important to underline that all governments, during the first phase of the pandemic, focused their attention on simply advising the use of masks, and only when the infection began to show its danger and uncontrollability did they resort to the formulation of laws, which more or less rigidly imposed the use of this device. Initially, there were no particular indications on the type of mask to wear and any type was fine, but when awareness of the extent of the problem increased, governments focused not only on the quantity, but also on the quality of filtering devices, directing the population towards models with better performance, such as FFP2/FFP3.

As the months went by, the dissemination of information on how to wear these devices in order to optimize their filtering effect became increasingly important.

During this second phase, it is less frequent to witness incorrect behaviors that nullify or drastically reduce the filtering power of the masks.

This study aims to verify if, during this advanced phase of the COVID-19 pandemic, the population changed the way they use and manage filter masks, given that the information on their use is widely spread worldwide [14,15,16,17].

During the use of masks, in addition to considering the way in which they are worn, it is also essential to focus attention on daily management.

As with all filtering devices, even masks can easily become infected, paradoxically becoming a vehicle for virus transmission. The risk of spreading the microorganisms trapped on the surface of this filter is linked, for example, to the continuous touching or adjusting the position of the mask itself; this leads to an unaware contamination of the hands with which other objects or surfaces are touched, which in turn become infected. For this reason, this research also investigated this important aspect, which is just as important as wearing the mask correctly [18]. 

## 2. Material and Method

This research was conducted in Italy (March 2022) and investigated the way in which the mask was worn and managed by the population.

The observation of the sample was carried out in large commercial establishments where the need to wear filter masks was particularly felt by people because the probability of contracting the infection is certainly greater in closed and crowded places. 

Particular attention was paid to go unnoticed during the observation period so as not to influence, in any case, the behavior of the subjects in the sample.

In this way all the observed people acted in an absolutely natural way, unaware of being watched.

The research was conducted over a 15-day period from 9:00 A.M. to 08:00 P.M.; in the choice of the representative sample, children were deliberately excluded because their “non-rational behavior” could distort the result. 

The number of subjects examined was 1084; the observation time for each individual subject was 60 s.

This study looked at various parameters, such as type of mask (surgical mask, FFP2/FFP3 with valve, FFP2/FFP3 without valve, and self-made or cloth-replaced mask), correct wearing (covering nose, mouth, and chin), and the correct management (touching or not the mask on the external surface). 

All data were collected by a single observer who, placed at a distance of about 5 m from the subject under observation, used pre-compiled forms, inserted into a smart phone, in which several fields were present. Each observed subject was assigned a sequential numerical code, which guaranteed anonymity; moreover, no photographs were ever taken in order to respect privacy. In each single module, the possible scenarios were already indicated, such as the various types of masks worn or if it was positioned correctly on the face and/or touched with the hands.

In this way, the observer only had to place a flag on the form; this facilitated both the collection of data and the fact that whoever collected them went completely unnoticed. 

In order to make the presence of the observer even more discreet, the latter after the observation period, equal to sixty seconds, moved to a distance in another area or another floor of the store, in order not to arouse suspects and undertake undisturbed observation on another research sample.

All collected data were statistically analyzed by chi-square test. 

## 3. Results

The observation of 1084 subjects in commercial activities showed that a large number of people wore filter masks (97.98%) and only a very small part of the sample (2.02%) did not protect their nose and mouth regardless of the danger of spreading the virus by air.

Among the subjects wearing masks, the preference was towards the FFP2/FFP3 type without a valve (93.72%), followed by the surgical type, even if only noticed on a fairly small number of people (2.95%). Even lower was the number of subjects who wore an FFP2/FFP3 with a valve (1.10%), and only a couple of people (0.18%) tried to self-build a protective mask.

As regards the ways in which the filter masks were worn by the sample, the following results are highlighted:

Among the subjects who wore surgical masks, it was found that many of them did it correctly (75%); only a few people (25%) made mistakes by not covering the nose, mouth, or chin, or leaving the mask loose on the face, thus reducing the filtering capacity.

People who wore FFP2/FFP3 masks without valves showed that they did it correctly in most cases (92.32%), and only a small number of subjects observed with this type of mask did not adequately cover their nose, mouth, and chin (7.67%).

The research also revealed that most of the people with FFP2/FFP3 masks with a valve wore the medical device appropriately (83.33%) and only a few subjects did not wear it correctly (16.66%).

Only two people were noticed with self-made masks and in both cases the “devices” were worn incorrectly (100%) (Table 1).

The study also reported data on the subjects' behavior towards worn filtering devices and the results are as follows:

Among the people who wore surgical masks, about half (53.12%) did not touch the medical device during the observation period; the other subjects (46.87%) did it at least once, and some of them repeatedly.

Even considering the people who wore FFP2/FFP3 without a valve, more than half of them (57.66%) did not touch the mask during the observation period, but a still large number of the sample (42.31%) did it once or more times.

The very few people who wore FFP2/FFP3 with a valve showed (58.33%) that they did not touch the mask, but a large number of them (41.66%) still touched the device with their hands.

Only two people wore self-made masks and both (100%) touched them even more times (Table 2).

## 4. Discussion

The filter masks, which are widely used in medicine and in all those fields in which it is necessary to filter the inhaled and exhaled air, represent an excellent means of containing the spread of microbes that spread through the air.

All medical devices must be accompanied by precise instructions on use and maintenance in order to fully exploit their potential. In this particular historical moment, where a medical device was imposed on the population, it emerged that informing is as important as imposing.

Our study showed that any type of filter mask must be worn and managed very carefully; otherwise, it completely loses its effectiveness [19,20,21].

At the beginning of when the COVID-19 pandemic started, the population was certainly not prepared to properly use a device that is normally intended for health personnel [22,23].

This happened because when governments began to spread information on masks, they could not imagine how much such a seemingly simple-to-use device needed detailed information on how to use it, what to do, and also what not to do in order to prevent a filter mask, which has the task of preventing infections, from turning into a vehicle of contagion instead. 

During the pandemic months, the compounding information played a decisive role in improving the management of these masks that seem of trivial usefulness, but which hide various criticalities and problems.

It is important to note that, during the second phase of the pandemic, the percentage of the sample who did not wear filter masks was really low (2.02%); this low percentage shows that the perception of the pandemic problem is very different from the initial period when general disorientation and misinformation prevailed. In fact, in the first months of the pandemic, a large part of the population was particularly skeptical even about the real existence of the infection, and in any case, the use of masks was sporadic [24]. 

The very low percentage of subjects observed with self-made masks (0.18%) confirms the greater diffusion of information among the population that basically understood that they cannot self-build a medical device, putting their own health and that of others at risk. At the beginning of the pandemic, in attempts to self-build masks, the population showed the greatest inspiration, but with particularly poor medical results; often, pieces of cloth with undoubted filtering capacity were simply used, literally placed on the face or tied in an approximate way or held in place with the hands in a discontinuous manner. This was the most striking demonstration of disinformation shown by the population [25,26].

Differently from our previous study, where the surgical masks were the most observed, in this new research, the data changed, and the most encountered masks were the FFP2/FFP3 without valves (93.72%), which represent the type suggested and/or imposed by the majority of European nations. This means that, for the same number of subjects wearing filter masks, a very high percentage of subjects with FFP2/FFP3 are much less likely to spread the virus [27].

Touching an infected mask with your hands could facilitate the contamination of other objects; all of this could happen, for example, with an infected and asymptomatic patient who, unaware of being infected with COVID-19, while correctly wearing a type of mask with a high filtering power such as FFP3, due to the breathing difficulties it could cause, repeatedly touches the device infecting his hands, which in turn become a means of spreading the virus [28,29]. 

As confirmation of how confused and imprecise the diffusion of the concepts of microbiology are in the population, it is interesting to note how almost all of the customers, observed inside the commercial shops, used the sanitizing gel provided at the entrance to reduce the microbial load on their hands; unfortunately the same subjects then repeatedly touched their potentially infected masks, probably unaware of having made useless the use of the disinfectant gel used a few minutes before [30].

All governments, in addition to giving indications on the type of mask to wear and how to use it correctly, probably should accompany their information with teachings of microbiology concepts that are easily accessible to the entire population.

This is not easy to do, because the level of basic biological knowledge can be very variable within the population, which is very uneven in all countries. These years of pandemic, therefore, put a strain on all the nations that tried, in various ways, to inform citizens in the best way, even if the strategic choices adopted and the information given to citizens did not always prove successful; to this is added that often within the same nation, the information released over time was discordant and misleading, therefore also losing credibility. The various strategic decisions, given by the various nations on the guidelines for citizens, also contributed to aggravating the disorientation [31].

## 5. Conclusions

The main enemy of this pandemic is an invisible microorganism; and the data in our possession confirm that it is particularly difficult to spread microbiological and medical information on infections to the “non-medical” population who are not used to fighting microscopic, and above all, invisible viruses capable of contaminating the air we breathe and the surfaces of the objects we live with on a daily basis.

This explains why even the people most attentive to wearing masks correctly that observed in our research often touched them without considering that these devices could potentially be infected, and therefore become a vehicle for transmission.

However, the study highlights how these years of pandemic made the population understand the importance of wearing proper masks, especially in closed and crowded places where the probability of spreading the virus is greater.

The high percentage of subjects identified as wearing FFP2/FFP3 masks confirms that choices on the type of mask to wear are also made on the basis of information widely disseminated by governments.

However, the data on the correct use of masks, which emerged from this study, indicate that the population needs some more information on how to manage this medical device.

The imposition of these masks and the dissemination by the government of correct information on how to wear them is a winning factor in the attempt to reduce the spread of the COVID-19 virus; but on the occasion of extraordinary events, such as the SARS-CoV-2 pandemic, information messages must be particularly clear, targeted, and repetitive without neglecting any aspect in order to reduce behavioral errors in the population. 

It should be emphasized that our research, while revealing a high percentage of subjects wearing appropriate filtering masks, shows that poor management of masks is still widespread in the population; in fact, both attempts to correct the wrong positioning of the device on the face and the need to move it continuously because it is annoying are still too frequent.

The key words, in situations such as those that the pandemic imposed on the entire planet, seem to be clarity and dissemination of certain information supported by scientific evidence in order to avoid useless and harmful misunderstandings in the population.

## Figures and Tables

**Table 1:** Distribution of result about wearing.

Correctness in Wearing	No Mask n (%)	Surgical Mask n (%)	FFP2/FFP3 No Valve n (%)	FFP2/FFP3 with Valve n (%)	Self Made Mask n (%)
	22 (2.02%)	32 (2.95%)	1016 (93.72%)	12 (1.10%)	2 (0.18%)
**Correct**	not applicable	24 (75%)	938 (92.32%)	10 (83.33%)	0 (0%)
**Incorrect**	not applicable	8 (25%)	78 (7.67%)	2 (16.66%)	2 (100%)

**Table 2:** Distribution of result about management.

Management	No Mask n (%)	Surgical Mask n (%)	FFP2/FFP3 No Valve n (%)	FFP2/FFP3 with Valve n (%)	Self Made Mask n (%)
	22 (2.02%)	32 (2.95%)	1016 (93.72%)	12 (1.10%)	2 (0.18%)
**Good** **management**	not applicable	17 (53.12%)	586 (57.66%)	7 (58.3%)	0 (0%)
**Bad** **managment**	not applicable	15 (46.87%)	430 (42.31%)	5 (41.6%)	2 (100%)

## Data Availability

Data supporting reported results can be asked to enzo.cumbo@unipa.it.
